# Motivational Factors and Barriers to Voluntary Blood Donation Among First-Time and Repeat Donors

**DOI:** 10.7759/cureus.101448

**Published:** 2026-01-13

**Authors:** Turki M Alanzi, Taha Alnazr, Dai Almutairi, Shahad Alanzi, Alaa Alghayib, Hind Almazyad, Shaden Althuwaini, Mesheal Alanazi, Shahad Alatawi, Razan Abusabah, Abdullah Alzahrani, Maryam Harshan

**Affiliations:** 1 Health Information Management and Technology, Imam Abdulrahman Bin Faisal University, Dammam, SAU; 2 General Practice, Prince Sultan Hospital, Eastern Health Cluster, Urayarah, SAU; 3 College of Medicine, University of Tabuk, Tabuk, SAU; 4 College of Pharmacy, University of Tabuk, Tabuk, SAU; 5 College of Pharmacy, King Saud University, Riyadh, SAU; 6 College of Medicine, Qassim University, Buraydah, SAU; 7 Medicine, King Saud Bin Abdulaziz University for Health Sciences, Riyadh, SAU; 8 College of Medicine, Batterjee Medical College, Jeddah, SAU; 9 Medical Laboratory Sciences, University of Tabuk, Tabuk, SAU; 10 College of Pharmacy, Umm Al-Qura University, Makkah, SAU; 11 College of Pharmacy, Al Baha University, Al Baha, SAU; 12 Hematology, Jazan University, Jazan, SAU

**Keywords:** deterrents, donor retention, first-time donors, motivation, saudi arabia, voluntary blood donation

## Abstract

Background: Voluntary blood donation is pivotal for healthcare systems, yet motivating first-time donors and retaining repeat donors remain challenging, particularly in middle-income countries like Saudi Arabia.

Aim: This study aimed to compare motivational and deterring factors between first-time and repeat blood donors in Saudi Arabia and to identify effective incentives to enhance donor participation.

Methods: A cross-sectional online survey was conducted with 427 adult blood donors (first-time and repeat) in Saudi Arabia using a validated 27-item questionnaire covering motivating factors, deterrents, and attractive incentives. Data were analyzed using descriptive statistics, t-tests, and chi-square tests.

Results: Repeat donors showed significantly higher motivation scores (mean altruism 4.02 vs. 2.99; p<0.0001) and lower deterrents related to fear and inconvenience compared to first-time donors (pain fear mean 2.05 vs. 3.61; p<0.0001). Both groups rated incentives such as snacks and branded items moderately but not significantly differently (p>0.05). Education influenced motivation and deterrence primarily among first-time donors.

Conclusion: Altruism and social influences drive sustained donation behavior, while fear and negative experiences limit first-time donor retention. Culturally tailored educational campaigns, community engagement, improved donor care, and involvement of religious and civic leaders are recommended to increase voluntary blood donation in Saudi Arabia.

## Introduction

Blood donation is one of the significant processes in the functioning of modern healthcare systems, which enables the treatment of various diseases and victims, such as trauma patients, patients undergoing surgeries, patients with hematologic disorders, and more. According to the World Health Organization (WHO), 118.5 million units of blood are collected per year globally, but only a small fraction of these is collected from the countries that have the greatest burden of need. Considering the blood donation rate, it is estimated that 31.5 donations per 1,000 people in high-income countries (HICs), 16.4 in upper-middle-income, 6.6 in lower-middle-income, and 5.0 in low-income countries (LICs), reflecting the large-scale disparity across the countries [1]. HICs, which account for only 16% of the world population, collect approximately 40% of all worldwide blood donations, reflecting the inequities in supply and demand in healthcare [2]. Considering the WHO recommendation of a minimum of 1% population to donate blood or 10 blood donations per 1000 population, many countries fall short of the recommended levels [3].

Focusing on Saudi Arabia, blood donation is considered to be a critical aspect in healthcare, but remains an underutilized resource. In 2011, it was assessed that approximately 1.46% of the Saudi population donated blood, reflecting only a minimum level of the WHO-recommended threshold [4]. The number of blood donors during 2010-2020 in governmental centers was estimated at ~325,847 ± 43,160, equivalent to about 13.8 donors per 1,000 population, which is relatively low compared to 32.6 per 1000 population in HICs [5,6]. These statistics reflect slow progress and continuing gaps in Saudi Arabia to ensure a sustainable and safe supply of blood.

In order to ensure a safe and sustainable blood supply, voluntary blood donation (VBD) is crucial. It enhances community solidarity and mitigates the risks associated with paid donors and replacement donors. It also ensures that the patients in emergencies or trauma, and chronic treatments, receive timely transfusions, especially for the economically weaker population [7-9]. In this context, first-time blood donation holds more significance, as it represents the entry point for generating a blood donor pool, transforming non-donors into active contributors who can be of great help during emergencies. It is of the utmost importance to provide encouragement and support to first-time donors, as pleasant initial experiences significantly improve the possibility of individuals becoming repeat donors, which in turn strengthens the long-term stability of the blood supply [10-14].

VBD may be influenced by various factors such as social motives, perceived costs, accessibility, etc., in various settings. Supporting the Theory of Planned Behavior (TPB), studies [15,16] in HICs have consistently revealed that intention (altruism), intrinsic motivators (self-esteem, citizenship), as well as extrinsic incentives (snacks, certificates, health checks), prosocial motivation, positive donor-site experience, and past behavior are the key factors influencing donation and re-donation. In addition, key deterring factors in HICs were found to be inconvenience and accessibility (time/location), poor staff interactions and adverse reactions, which have significantly affected repeat donations [17-21]. Among the first-time and young donors, fear of needles, pain, and adverse reactions were identified to be the main deterring factors, which can significantly affect return rates if not managed [22].

In relation to middle-income countries (MICs), social cues (being asked, peers/family donors), trust in services, and personal health benefits were identified to be the key motivating factors. For example, studies [23-25] from MICs like Saudi Arabia and India identified urge to help, awareness, and realization of need, social referrals to be the key motivating factors, while deterring factors included lack of awareness, fear, and misconceptions. In this context, it can be understood that knowledge gaps, social cues, and the urge to help more strongly predict intent to donate [26]. Furthermore, cultural beliefs and the spread of misinformation, such as losing strength or getting diseases due to blood donation, affect intention to donate blood; while a poor blood donation management system and infrastructure affect repeat donation [25,27,28].

In relation to LICs, it has been observed that altruism coexists with instrumental motives (e.g., health checks) and structural barriers such as lack of awareness of donation sites, transport/time constraints, and concerns about blood being sold [26,29,30]. In addition to fear, poor knowledge, ineffective incentives, poor infrastructure, and service experts, lack of established infrastructure was identified to be a major deterrent factor for blood donation [30]. It has also been observed that willingness to donate blood rises when a known person personally invites for blood donation, reflecting the lack of trust in blood donation organizers [31].

Focusing on the incentive strategies, different incentives reflected different impacts on the intention to donate blood in the previous studies. It was observed that incentives like a single gift card would significantly increase the donors' turnout in a short period, but single health incentives (e.g., cholesterol) offers do not increase the donors' turnout rate [32]. However, health test bundles or comprehensive health tests that include multiple biomarkers as an incentive raised return rates and reflected a meaningful and credible benefit. Improving operations such as extended hours for collection, mobile drives, shorter waiting period, supportive staff can significantly improve donor experience and directly target the key deterrent factor - inconvenience [33,34]. Furthermore, psychophysiological strategies such as pre-hydration, applied muscle tension, and fear screening can reduce reactions and provide a safe and reliable experience for first-time donors [35]. In addition, behavioral (TPB-based) strategies such as messaging, feedback on impact, and fairness/reciprocity frames led to re-donation and improved relationship quality between donors and receivers [36].

The success of intervention strategies mainly depends on contextual factors, which can be identified from the different motivating and deterring factors and incentives across the regions. For instance, effective and efficient donor management, regular reminders, and educational campaigns are a few key strategies in HICs; whereas, school/university drives, community engagement, and mobilizing social networks are a few strategies adopted in MICs; and public sensitization, decentralization of donation centers, and infrastructure improvements strategies are adopted in LICs [37-39].

Given the importance of voluntary and first-time blood donors, it is important to understand the motivating and deterring factors in blood donation. Motivating factors include altruistic, social, personal-benefit, and environmental cues that stimulate blood donation [40,41]. On the other hand, discouraging factors refer to perceived barriers that discourage donation. These barriers can include fear, inconvenience, a lack of information, or unfavorable experiences from the past [42,43]. Taking into consideration these imperatives, the purpose of the current study is to investigate and compare the factors that motivate, the factors that discourage, and the perceptions of attractive incentives for blood donation among first-time donors in Saudi Arabia as opposed to repeat donors. The ultimate goal of this research is to identify actionable levers that can be used to increase voluntary donation participation and retention. Accordingly, the objectives of this study include the following: to identify and compare the motivating and deterring factors influencing VBD among first-time and repeat donors; to assess the perceptions of attractive incentives that encourage blood donation participation and retention among both donor groups; to provide evidence-based recommendations for enhancing donor recruitment, improving retention strategies, and strengthening VBD programs.

## Materials and methods

Study setting and participants

This study adopted a cross-sectional survey design. The study was conducted in Saudi Arabia for eight weeks between 1 July and 27 August 2025. The study targeted adult individuals aged 18 years and above who had donated blood at least once, encompassing both first-time and repeat donors. Participants were recruited from blood donation centers, mobile blood drives, and online social media channels related to blood donation.

Sampling

This study employed a non-probability, mixed recruitment approach using convenience sampling [44]. The sampling frame comprised adult individuals (≥18 years) residing in Saudi Arabia who had donated blood at least once, including both first-time and repeat donors. Participants were recruited from blood donation centers and mobile blood drives, as well as through online platforms and social media channels related to blood donation. This recruitment strategy was adopted to capture a diverse pool of donors across different regions within the study period; however, it did not constitute true random sampling. Sample size estimation was guided by Cochran’s formula, indicating a minimum requirement of 385 participants. A total of 427 eligible respondents completed the survey and were included in the final analysis.

Questionnaire design

Data were collected using a structured, self-administered questionnaire adapted from the validated instrument developed by Sharifah et al. [45] for assessing motivating and hindering factors for blood donation. The tool comprised 27 items distributed across three domains, which include: (1) Motivating Factors (12 items) - measuring altruism, social influence, health beliefs, and emotional satisfaction (e.g., “Donating blood helps others in need,” “I feel proud when I donate”); (2) Deterring Factors (eight items) - evaluating perceived barriers such as fear of pain, inconvenience, and adverse experiences (e.g., “Donating blood is painful,” “I felt unwell after donating”); (3) Attractive Incentives (seven items) - assessing the appeal of various rewards and recognition mechanisms (e.g., snacks, certificates, vouchers).

Responses were recorded on a five-point Likert scale ranging from 1 = Strongly Disagree to 5 = Strongly Agree. To ensure linguistic and cultural validity, the questionnaire was translated into Arabic and reviewed by two bilingual experts in public health and behavioral research. Minor adjustments were made for cultural relevance and clarity. A pilot study involving 20 donors was conducted to assess the instrument’s reliability and comprehensibility. Although individual Likert-scale items are ordinal in nature, the present study treated aggregated domain scores (motivating factors, deterring factors, and incentive perceptions) as approximately continuous variables for analytical purposes. This approach is supported by previous studies [46-48] indicating that summed or averaged Likert-scale scores with five or more response categories and adequate internal consistency can be reasonably analyzed using parametric methods. In the current study, each domain demonstrated good internal reliability (Cronbach’s α = 0.739-0.832) [49], and domain scores were computed by averaging multiple related items, thereby approximating continuous measurement.

Data analysis

Data were coded and analyzed using IBM SPSS Statistics for Windows, Version 22 (Released 2013; IBM Corp., Armonk, New York, United States). Descriptive statistics (means, standard deviations, frequencies, and percentages) summarized participant demographics and individual item responses. To assess differences between first-time and repeat donors, independent samples t-tests were performed for continuous variables and chi-square tests for categorical data.

Ethical considerations

The study adhered to ethical principles outlined in the Declaration of Helsinki. Ethical approval was obtained from the Institutional Review Board of the College of Public Health, Imam Abdulrahman Bin Faisal University (Ref: IRB-2025-03-0434). All participants were informed about the study’s objectives, confidentiality, and their right to withdraw at any stage without consequences on the first page of the online survey, where the participants proceeded after giving their consent. In addition, no identifying information was collected to preserve participant anonymity.

## Results

Table 1 shows that most participants (114, 26.7%) were aged 45-54 years, with a slight male majority (226, 52.9%). The mean age of participants was 39.5 years (SD = 12.5). The sample was diverse in occupation, led by 93 (21.8%) self-employed and 90 (21.1%) students. Educationally, most participants, including 167 (39.1%), held a bachelor’s degree, followed by 92 (21.6%) diploma holders and 83 (19.4%) primary/secondary level participants.

**Table 1:** Participants details

Variables	Sub-variables	N	Relative frequency
Age (in years)	18-24	73	17.10%
	25-34	84	19.67%
	35-44	105	24.59%
	45-54	114	26.70%
	>54	51	11.94%
Gender	Male	226	52.93%
	Female	201	47.07%
Occupation	Employed	83	19.44%
	Retired	84	19.67%
	Self-employed	93	21.78%
	Student	90	21.08%
	Unemployed	77	18.03%
Education	Primary/Secondary	83	19.44%
	Diploma	92	21.55%
	Bachelor’s	167	39.11%
	Postgraduate	70	16.39%
	Doctoral	15	3.51%

Table 2 reveals significant differences in motivational factors between first-time and repeat blood donors. The largest mean differences were observed for altruistic motives, including the desire to help others (mean difference = +1.03) and awareness of blood shortages (mean difference = +1.05), indicating markedly stronger prosocial orientation among repeat donors. Social reinforcement factors, such as peer and family influence, also showed notable differences, with repeat donors reporting consistently higher engagement. Overall, the results suggest that repeat donors are significantly more motivated by altruism, social reinforcement, and personal satisfaction than first-time donors.

**Table 2:** Differences between first-time and repeat donors in relation to motivating factors for blood donation using Pearson t-test * Statistically significant at p < 0.05.

Factors	First-time donors		Repeat donors		T-value	P-value
	Mean	Standard deviation	Mean	Standard deviation		
I want to create a good practice/charity/help others.	2.99	0.93	4.02	0.66	12.0844	<0.0001*
Donating blood makes me feel like a hero.	3.11	0.79	4.00	0.67	11.6371	<0.0001*
There is a shortage of blood supply to people in need.	3.00	0.86	4.05	0.65	12.9908	<0.0001*
Donating blood is good for my health.	3.02	0.84	4.01	0.66	12.4295	<0.0001*
My friends/colleagues donate blood.	2.90	0.84	4.01	0.60	14.3186	<0.0001*
Lecturers/staff at my university or college donate blood.	2.99	0.91	3.97	0.65	11.6988	<0.0001*
Someone in my family is a blood donor.	3.06	0.87	3.90	0.64	10.3551	<0.0001*
Someone will be proud of me if I donate blood.	3.02	0.87	3.93	0.68	11.0995	<0.0001*
I am interested in blood donation campaigns promoted on social media, flyers, etc.	3.10	0.86	3.99	0.67	10.8393	<0.0001*
I love the atmosphere/good conditions in the blood donation area/among staff and nurses on duty.	2.90	0.79	3.97	0.65	14.1487	<0.0001*
I want the reward/incentive.	2.89	0.88	3.99	0.62	13.5788	<0.0001*
I would feel bad if I do not donate.	3.09	0.76	3.99	0.68	12.1356	<0.0001*

Table 3 shows significant differences between first-time and repeat donors across all deterring factors. First-time donors reported considerably higher levels of perceived barriers compared with repeat donors across all deterring factors. The most pronounced mean differences were related to fear of pain (mean difference = +1.56), post-donation discomfort (mean difference = +1.61), and perceived inconvenience related to time and location (mean difference = +1.63). These differences indicate that psychological and experiential concerns are substantially more salient among first-time donors, whereas repeat donors reported consistently low levels of deterrence, reflecting greater familiarity and confidence with the donation process.

**Table 3:** Differences between first-time and repeat donors in relation to deterring factors for blood donation using Pearson t-test * Statistically significant at p < 0.05.

Factors	First-time donors		Repeat donors		T-value	P-value
	Mean	Standard deviation	Mean	Standard deviation		
Donating blood is painful.	3.61	0.94	2.05	0.77	17.4501	<0.0001*
Donating blood is troublesome (e.g., time and location).	3.59	0.95	1.96	0.74	18.0322	<0.0001*
I do not like to see blood.	3.47	0.98	2.10	0.81	14.5754	<0.0001*
Donating blood takes a long time.	3.51	0.97	2.04	0.79	15.9451	<0.0001*
I dislike skipping class to donate blood.	3.43	0.96	2.04	0.79	14.9777	<0.0001*
I felt worse after donating blood (light-headedness, nausea, headache, blackouts/fainting, etc.).	3.66	0.85	2.05	0.80	18.9281	<0.0001*
Nurse/staff is not friendly.	3.59	0.98	1.99	0.76	17.3496	<0.0001*
Incentives are not attractive.	3.53	0.87	2.12	0.82	16.3658	<0.0001*

Table 4 indicates that there were no statistically significant differences between first-time and repeat donors in their perceptions of attractive incentives. Both groups showed moderate interest across all incentive types, with mean scores ranging from approximately 3.4 to 3.6. The most appealing incentives for both groups included snacks (3.56 vs. 3.44), logo-branded items (3.55 vs. 3.48), and entertainment tickets (3.53 vs. 3.48). Repeat donors rated acknowledgments and vouchers slightly higher, suggesting some appreciation for recognition and tangible rewards. Overall, the findings suggest that while incentives are generally viewed favorably, they play a secondary role compared to motivational or psychological factors in influencing blood donation behavior.

**Table 4:** Differences between first-time and repeat donors in relation to attractive incentives for blood donation using Pearson t-test

Factors	First-time donors		Repeat donors		T-value	P-value
	Mean	Standard deviation	Mean	Standard deviation		
Movie ticket/Amusement Park ticket/Bowling ticket	3.53	1.11	3.48	1.13	0.7914	0.6618
Biscuits/titbits/free snacks after donating	3.56	1.06	3.44	1.12	1.0568	0.2838
Items that have the logo of the blood donation centre, such as T-shirts, towels, mugs or bags	3.55	1.11	3.48	1.13	0.6224	0.5403
Acknowledgments/credit to total hours of community service performed/merit points	3.46	1.07	3.58	1.10	1.1337	0.2795
A chance to gain exemptions/skipping class	3.49	1.05	3.44	1.13	0.4485	0.6560
Shopping or food vouchers	3.40	1.11	3.53	1.11	1.1347	0.2458
Credits for prepaid mobile phone	3.47	1.19	3.51	1.09	0.3953	0.7269

Table 5 compares motivating factors across demographic groups among first-time and repeat donors using analysis of variance (ANOVA). Results show no significant gender or age differences in motivation for either group. However, a significant difference by education level was observed among first-time donors, with those holding diplomas and postgraduate degrees reporting slightly higher motivation (means: 3.06 and 3.08) compared to those with primary/secondary education (2.90). Among repeat donors, motivation levels remained uniformly high across all educational backgrounds (mean ≈ 3.97-4.01). Overall, the findings suggest that education influences motivation primarily among first-time donors, while repeat donors maintain consistently strong motivation regardless of demographic factors.

**Table 5:** Differences among the first-time and repeat donors with respect to motivating factors using ANOVA * statistically significant at p < 0.05; ** analyzed using the Pearson t-test. ANOVA: analysis of variance

Variables	Sub-variables	First-time donors					Repeat donors				
		N	Mean	Variance	F-value/T-value**	P-value	N	Mean	Variance	F-value/T-value**	P-value
Gender	Male	75	2.98	0.06	1.9763**	0.1799	151	3.97	0.04	1.9687**	0.1201
	Female	73	3.03	0.05			128	4.00	0.03		
Age (in years)	18-24	35	2.98	0.04	1.3849	0.2421	38	4.00	0.05	0.2218	0.9261
	25-34	24	2.99	0.08			60	3.98	0.04		
	35-44	32	2.99	0.05			73	3.97	0.04		
	45-54	37	3.08	0.05			77	3.99	0.03		
	>54	20	2.96	0.06			31	3.98	0.03		
Education	Primary/Secondary education	27	2.90	0.04	2.5996	0.0386*	56	4.01	0.04	0.5515	0.6981
	Diploma	37	3.06	0.06			55	3.99	0.03		
	Bachelor’s	55	2.98	0.05			112	3.97	0.04		
	Postgraduate	23	3.08	0.05			47	3.99	0.03		
	Doctoral	6	3.03	0.06			9	4.00	0.05		

Table 6 shows that deterring factors did not significantly differ by gender or age among either first-time or repeat donors. However, a significant difference by education level was found among first-time donors (p = 0.0057), with those holding diplomas (mean = 3.68) and primary/secondary education (3.63) reporting stronger deterrents compared to postgraduate (3.42) and doctoral (3.44) participants. Among repeat donors, deterrent scores remained uniformly low (mean ≈ 2.0) across all education levels. These results suggest that lower educational attainment is associated with greater fear or perceived barriers to donation among first-time donors, while repeat donors exhibit consistently reduced deterrence regardless of demographic factors.

**Table 6:** Differences among the first-time and repeat donors with respect to deterring factors using ANOVA * statistically significant at p < 0.05; ** analyzed using the Pearson t-test. ANOVA: analysis of variance

Variables	Sub-variables	First-time donors					Repeat donors				
		N	Mean	Variance	F-value/T-value**	P-value	N	Mean	Variance	F-value/T-value**	P-value
Gender	Male	75	3.58	0.12	1.9765**	0.2559	151	2.04	0.09	1.9685**	0.7321
	Female	73	3.52	0.09			128	2.05	0.07		
Age (in years)	18-24	35	3.52	0.12	1.0678	0.3747	38	2.05	0.08	0.0599	0.9933
	25-34	24	3.64	0.12			60	2.05	0.09		
	35-44	32	3.57	0.10			73	2.05	0.08		
	45-54	37	3.48	0.07			77	2.04	0.08		
	>54	20	3.58	0.12			31	2.03	0.09		
Education	Primary/Secondary education	27	3.63	0.07	3.7949	0.0057*	56	2.05	0.08	0.4523	0.7706
	Diploma	37	3.68	0.07			55	2.01	0.09		
	Bachelor’s	55	3.49	0.11			112	2.07	0.09		
	Postgraduate	23	3.42	0.13			47	2.02	0.06		
	Doctoral	6	3.44	0.02			9	2.03	0.17		

## Discussion

This study provides valuable insights into motivational and deterring factors influencing VBD among first-time and repeat donors in Saudi Arabia. The results reveal significant differences between these groups, highlighting both consistent patterns and context-specific nuances that align with global literature.

Consistent with prior studies in HICs, altruistic motives such as the desire to help others and address blood shortages strongly motivated repeat donors, who exhibited higher intrinsic and extrinsic motivation scores across all factors compared to first-time donors [19,20]. This corroborates the TPB, emphasizing altruism and self-identity as key predictors of donor retention [40]. The finding that social influence from peers and family was more pronounced among repeat donors also aligns with previous research showing the importance of social reinforcement in repeat donation.​

Fear and perceived barriers remained the most significant deterring factors, particularly among first-time donors, who reported greater apprehension regarding pain, adverse reactions, and inconvenience. The higher fear scores and negative perceptions towards staff friendliness underscore psychological and experiential barriers documented in both HICs and MICs, such as Saudi Arabia and India [26-29]. These barriers reflect ongoing challenges in donor recruitment and retention strategies identified in earlier studies [29-32]. The lower deterrence among repeat donors suggests habituation and increased trust with experience, supporting intervention models targeting first-time donor reassurance [42,50].​

The moderate and statistically similar perceptions of incentives among both donor groups indicate that while rewards such as snacks and branded items are liked, they likely play a secondary role to intrinsic motivators and barrier reduction. This aligns with mixed evidence from prior research showing that simple, non-health-related incentives may boost short-term turnout but have limited long-term effect without addressing psychological and logistical concerns [36].​

The demographic analysis highlights education as a significant correlate, with lower educational attainment associated with stronger deterrence and lower motivation among first-time donors. This finding echoes similar observations from MIC contexts emphasizing knowledge and awareness gaps as critical impediments to initial donation. Efforts targeting educational enhancement and tailored communication for less educated donor segments could prove effective [51,52].​

Several contextual factors unique to Saudi Arabia may influence these dynamics, such as cultural beliefs and limited public awareness, as suggested in previous literature. The importance of community engagement, religious leadership advocacy, and culturally appropriate messaging emerges as vital for increasing donor pools, echoing successful interventions in MICs and LICs [41-43]. Moreover, infrastructural improvements, efficient donor care, and psychophysiological strategies to reduce adverse reactions are supported by evidence as ways to enhance donor experience and retention.

Theoretical and practical implications

This study reinforces and extends prominent psychological theories such as the TPB and Self-Determination Theory (SDT) within the context of VBD. The findings emphasize the importance of intrinsic motivation, particularly altruism and social relatedness, which align with SDT’s assertion that fulfilling basic psychological needs of competence, autonomy, and relatedness fosters sustained behavior. The differential motivational profiles between first-time and repeat donors highlight critical nuances in donor identity development, supporting TPB’s focus on intention formation influenced by attitudes and social norms. Practically, these insights underscore the necessity of tailored intervention strategies that enhance first-time donors’ positive experiences to foster internalized motivation and transition to repeat donation. Addressing psychological deterrents such as fear and inconvenience through supportive donor care and accessible donation services can reduce barriers, as supported by evidence from MIC and LIC settings. Furthermore, culturally sensitive messaging and community engagement are imperative to bridge educational gaps and counter prevailing myths about blood donation, particularly in societies where misinformation influences donor behavior. Overall, integrating behavioral theory with practical donor management enables blood programs to design evidence-based, context-specific strategies that promote sustainable VBD, critical for ensuring blood supply equity and safety globally.

Recommendations

To enhance VBD in Saudi Arabia, recommendations should include the deployment of motivational messages tailored to cultural values, leveraging the country's strong religious context, where blood donation is perceived as a religious duty by the majority. Non-monetary incentives like token gifts and formal recognition, such as medals for repeat donations, should be widely publicized to build donor loyalty. Community education campaigns targeting misconceptions and knowledge gaps are crucial, especially through schools, universities, and social media platforms, to address fears and encourage first-time donations. Improving donor care by ensuring friendly and supportive staff interactions, minimizing inconvenience by extending donation hours, and providing pre- and post-donation follow-ups will improve donor experience and retention. Engaging religious and community leaders as advocates can amplify messaging and reinforce donation as a moral and social responsibility aligned with Saudi values. Additionally, mobile blood donation units in workplaces and universities, alongside integration of digital platforms like the "Sehhaty" app for ease of appointment scheduling and communication, offer innovative solutions to reduce access barriers and engage younger donors increasingly through technology. Psychophysiological strategies such as pre-donation hydration and fear screening may further improve donor comfort and safety perceptions. These combined, culturally sensitive, and technology-enabled strategies align with Saudi Arabia’s Vision 2030 goals for a sustainable, VBD system and can serve as a model for similar middle-income contexts.​

Limitations

This study has several limitations that should be considered when interpreting the findings. First, the use of a self-administered, self-reported questionnaire may have introduced social desirability and recall bias, particularly for motivation-related items such as altruism and moral obligation. As a result, motivational scores, especially among repeat donors, may be overestimated, while deterrent factors may be underreported, potentially inflating observed differences between donor groups. Second, recruitment through online platforms and blood donation centers using a convenience-based approach may have led to selection bias, favoring individuals who are more engaged with blood donation services, more health-conscious, or digitally literate. This may partially explain the relatively high motivation and low deterrence observed among repeat donors, and limits the generalizability of the findings to less engaged, infrequent, or technologically marginalized populations. Third, the cross-sectional design captures donor perceptions at a single point in time and does not account for changes in motivation or deterrence across donation experiences. Consequently, causal interpretations regarding the transition from first-time to repeat donation cannot be made. Finally, the reliance on broad age categories and the absence of clinical or procedural variables (e.g., venous access difficulty or deferral history) may have obscured factors that differentially affect donor experience, particularly among subgroups such as female or older donors. Together, these limitations suggest that the findings should be interpreted as indicative of associations rather than causal mechanisms, and highlight the need for longitudinal and mixed-methods research to further validate and extend the results.

## Conclusions

This study highlights important differences between first-time and repeat voluntary blood donors in Saudi Arabia, emphasizing the critical role of altruistic motivation and social influences in fostering donor retention. The identification of fear, inconvenience, and negative experiences as primary deterrents among new donors underscores the need for targeted strategies to create positive first donation experiences. While incentives are positively received, intrinsic factors remain more influential, suggesting strategies should prioritize education, community engagement, and improved donor care. Recommendations for Saudi Arabia include culturally tailored motivational messaging, non-monetary recognition, community and educational campaigns, enhanced donor support, and active involvement of religious and civic leaders. Integrating digital platforms and mobile services can further facilitate donor access and engagement. Despite limitations such as online survey bias and cross-sectional design, the findings provide actionable insights to advance sustainable blood donation programs aligned with Vision 2030 goals, with potential applicability to similar contexts worldwide.
